# p62/Sqstm1 rescue in muscle retards the progression of steatohepatitis in *p62/Sqstm1*-null mice fed a high-fat diet

**DOI:** 10.3389/fphys.2022.993995

**Published:** 2022-11-01

**Authors:** Ikuru Miura, Kosuke Okada, Akiko Ishii, Eiji Warabi, Takahisa Watahiki, Keii To, Hitoshi Shimano, Shunichi Ariizumi, Junichi Shoda

**Affiliations:** ^1^ Doctoral Program in Sports Medicine, Graduate School of Comprehensive Human Sciences, University of Tsukuba, Tsukuba, Japan; ^2^ Department of Medical Science, Faculty of Medicine, University of Tsukuba, Tsukuba, Japan; ^3^ Department of Internal Medicine (Neurology), Faculty of Medicine, University of Tsukuba, Tsukuba, Japan; ^4^ Laboratory Animal Resource Center in Transborder Medical Research Center, and Department of Anatomy and Embryology, Faculty of Medicine, University of Tsukuba, Tsukuba, Japan; ^5^ Doctoral Program in Clinical Sciences, Graduate School of Comprehensive Human Sciences, University of Tsukuba, Tsukuba, Japan; ^6^ Doctoral Program in Medical Sciences, Graduate School of Comprehensive Human Sciences, University of Tsukuba, Tsukuba, Japan; ^7^ Department of Internal Medicine (Endocrinology and Metabolism), Faculty of Medicine, University of Tsukuba, Tsukuba, Japan; ^8^ From the Department of Surgery, Institute of Gastroenterology, Tokyo Women’s Medical University, Shinjuku, Japan

**Keywords:** p62/SQSTM1, obesity, skeletal muscle, insulin resistance, non-alcoholic steatohepatitis

## Abstract

**Introduction:** Obesity is a risk factor for many diseases because it leads to a reduction in skeletal muscle mass and promotes insulin resistance. *p62/Sqstm1*-knockout mice are a model of metabolic syndrome; show obesity, insulin resistance, and non-alcoholic fatty liver (NAFL); and develop non-alcoholic steatohepatitis (NASH) in response to the feeding of a high-fat diet (HFD). These phenotypes suggest that muscle p62 may prevent obesity-induced muscle dysfunction. In the present study, we aimed to determine the effects of muscle p62 on skeletal muscle mass, muscle strength, insulin resistance, and NASH pathology.

**Methods:** We generated muscle-specific *p62* gene rescue mice (*p62*-mRes), which express p62 only in muscle and were derived from *p62*-knock out mice (*p62* ^*KIKI*^) using the *cre/loxp* system. *p62* ^*KIKI*^ and *p62*-mRes mice were fed an HFD for 20 weeks and their phenotypes were compared.

**Results:** HFD-feeding caused severe obesity in both *p62* ^*KIKI*^ and *p62*-mRes mice, but there was no effect of muscle p62 on body mass. Limb skeletal muscle mass, grip strength, and the cross-sectional area of muscle fibers were higher in *p62*-mRes mice than in *p62* ^*KIKI*^. The glucose tolerance and insulin sensitivity of the *p62*-mRes mice were also superior. The protein expression of mechanistic target of rapamycin, which promotes muscle protein synthesis, and GLUT4, a glucose transporter in skeletal muscle, were higher in the *p62*-mRes mice. *p62* ^*KIKI*^ mice developed severe NASH when fed an HFD, but the progression of NASH was retarded by *p62* gene rescue in muscle, and the expression of *Tgf-β1*, which encodes a factor that promotes hepatic fibrosis, was reduced.

**Conclusion:** Rescue of muscle-specific *p62* in the whole-body *p62* knock-out mice ameliorates the insulin resistance and retards the progression of NASH caused by systemic *p62* ablation.

## Introduction

Obesity is a serious social problem, and in 2016 the World Health Organization reported that approximately 39% of adults were overweight worldwide. In obesity, an excessive accumulation of adipocytes results in greater secretion of certain hormones and proinflammatory cytokines (adipokines), induces chronic inflammation, and promotes insulin resistance (Kahn et al., 2006; Shoelson et al., 2006). The failure of glucose homeostasis associated with insulin resistance increases the risks of several diseases, including type 2 diabetes mellitus (T2DM), dyslipidemia, and non-alcoholic fatty liver disease (NAFLD) (Kodama et al., 2014; Younossi, 2019). NAFLD is a generic term for the most common chronic liver disease (Younossi, 2019; Loomba et al., 2021), which involves the accumulation of fat in the liver, and the number of patients with NAFLD is increasing rapidly alongside the increase in the prevalence of obesity. As part of the spectrum of this disease, non-alcoholic steatohepatitis (NASH), which is characterized by liver inflammation and fibrosis, is a serious disease that can progress to cirrhosis and hepatocellular carcinoma. Because obesity-induced insulin resistance is fundamental to the development and progression of NASH, it is important to prevent this.

Skeletal muscle is the largest organ in the body and it has roles in locomotor function and posture maintenance, but also in whole-body metabolism. Skeletal muscle is a key target of insulin, and approximately 40% of the glucose ingested is taken up by this tissue and stored as glycogen (Argiles et al., 2016). There are close relationships between obesity, T2DM, and skeletal muscle mass. A previous cohort study (Hong et al., 2017) showed that the incidence of T2DM during the follow-up period was higher in participants with low skeletal muscle mass index (SMI; total skeletal muscle mass/body mass (kg/kg) × 100). Furthermore, in our previous study, we performed a quartile analysis using an index of sarcopenic obesity, the SV ratio (skeletal muscle mass/visceral fat area (g/cm^2^), which showed that participants in the group with the lowest SV ratio had the highest levels of insulin resistance, hepatic fat accumulation, and hepatic inflammation (Shida et al., 2018). In contrast, the increase in skeletal muscle mass induced by resistance training causes an improvement in insulin resistance (Bucci et al., 2016). The maintenance of skeletal muscle mass and function plays an important role in the management of insulin-glucose metabolism in obesity.

Insulin binds to its receptor, a transmembrane protein, which promotes the phosphorylation of AKT, leading to the translocation of the glucose transport GLUT4 to the sarcolemma and an increase in glucose uptake into muscle (Zierath et al., 2000). In insulin resistance, defects in this signal transduction reduce insulin-stimulated glucose uptake (Guo, 2014). Insulin also regulates skeletal muscle mass. Insulin activates mechanistic target of rapamycin complex 1 (mTORC1) via AKT (Bodine et al., 2001; Rommel et al., 2001), leading to the activation of factors that induce protein synthesis, such as p70S6K, and inhibits muscle atrophy through an effect on 4EBP-1(Glass, 2005). These effects mediate an increase in skeletal muscle mass. Additionally, AKT inhibits muscle atrophy by reducing the expression of MuRF-1 and atrogin-1 *via the* phosphorylation of FoXO3/4 (Glass, 2005). Furthermore, compensatory hyperinsulinemia occurs in insulin resistance, and there is a positive relationship between the circulating concentrations of insulin and myostatin, which promotes muscle atrophy (Tanaka et al., 2018). Thus, in insulin resistance, which is associated with both obesity and T2DM, a decrease in muscle synthesis and an increase in muscle degradation occur, which results in skeletal muscle atrophy.

Mice with a deletion of *p62/Sqstm1* (*p62*-KO), a regulator of selective autophagy, show hyperphagia-induced obesity owing to abnormal leptin signaling, and develop a phenotype similar to that of metabolic syndrome in humans (Okada et al., 2009; Harada et al., 2013), including glucose intolerance, insulin resistance, and non-alcoholic fatty liver (NAFL). In addition, *p62*-KO mice develop severe obesity and non-alcoholic steatohepatitis (NASH) when fed a high-fat diet (Duran et al., 2016; Miura et al., 2021). It has previously been shown that p62 is involved in the progression of human hepatocellular carcinoma (Umemura et al., 2016), suggesting that p62 also plays a role in liver pathology in humans. In the skeletal muscle of aging mice, excessive accumulation of p62 has been observed (Sakuma et al., 2016), which suggests that disordered autophagy is involved in age-related muscle atrophy (sarcopenia), and it has been suggested that the loss of p62 function may promote skeletal muscle atrophy. The phenotype of the *p62*-KO mice also suggests that skeletal muscle p62 helps minimize obesity-related glucose intolerance and insulin resistance. However, to our knowledge, the effects of muscle p62 on obesity-induced glucose intolerance, insulin resistance, and NASH have not been investigated.

In the present study, we aimed to assess the role of p62 in muscle by generating muscle-specific *p62* gene rescue mice (*p62*-mRes), in which p62 is only expressed in muscle. We determined the effects of p62 expression in muscle on skeletal muscle mass and function, glucose intolerance, insulin resistance, and NASH pathology in HFD-fed mice.

## Materials and methods

### Animals

**FIGURE 1:**
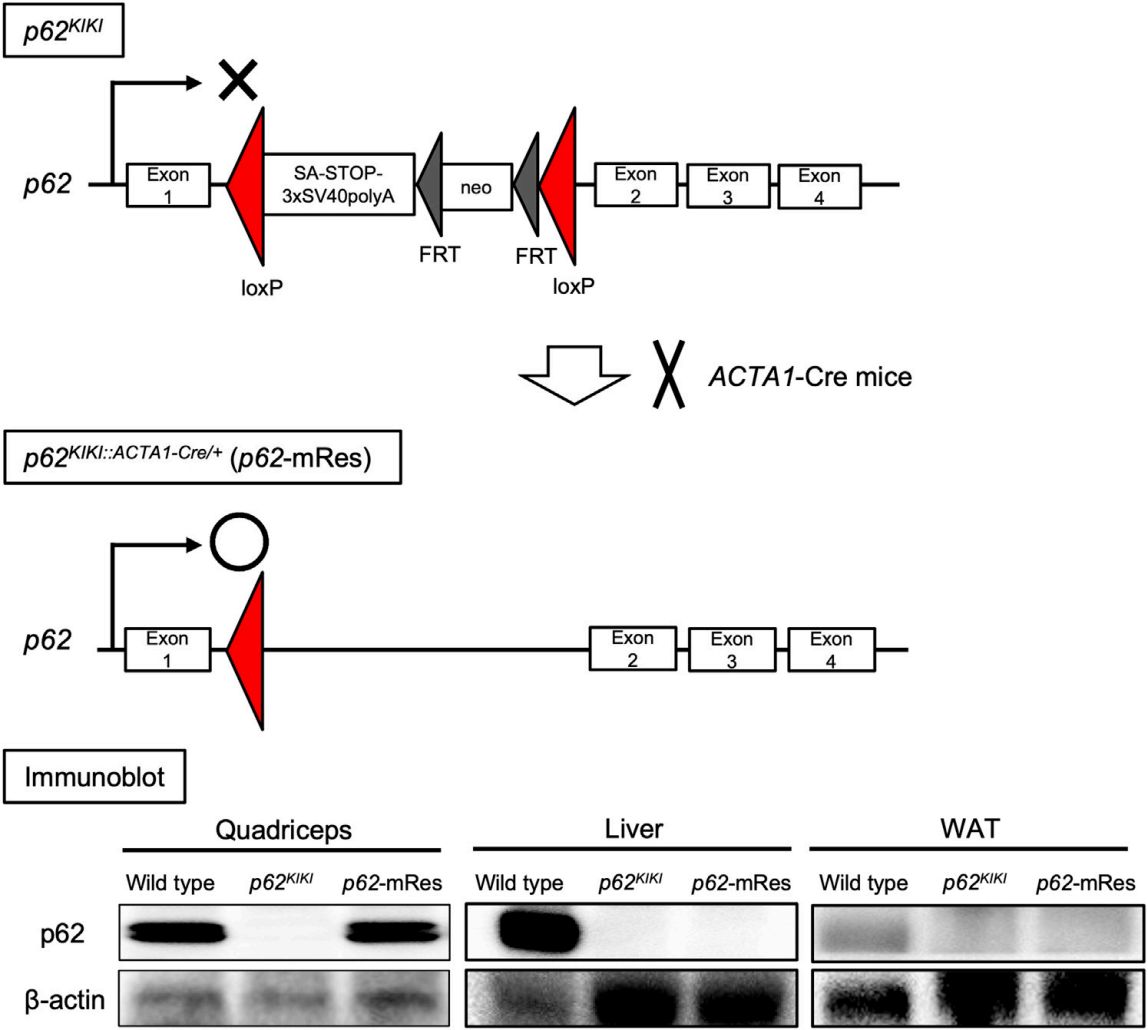
Generation of muscle-specific *p62* gene rescue mice. Schematic diagram of the design of the *p62* knock-in gene (*p62* ^*KIKI*^) and the procedure for generating muscle-specific *p62* gene rescue mice (*p62*-mRes). *p62*-mRes mice were generated by crossing with human alpha-skeletal actin (*ACTA1*)-Cre mice. *p62* gene rescue in muscle was confirmed by immunoblotting of *quadriceps*, liver, and white adipose tissue (WAT).

Mice on C57BL/6 background were used. We generated *p62*-knock-in mice (*p62* ^*KIKI*^), in which a transcription termination signal flanked by *loxp* sequences and a *polyA-FRT-neomycin resistance gene cassette (neo)-FRT* sequence were inserted into the intron between exons 1 and 2 of the *p62* gene (Figure 1). These mice have whole-body *p62* deficiency. Muscle-specific *p62* gene rescue mice (*p62* ^*KIKI:ACTA1-Cre/+*^: *p62*-mRes) were obtained by crossing *p62* ^*KIKI*^ and human alpha-skeletal actin (*ACTA1*)-Cre mice using the *cre/loxp* system (Sauer, 1987). The mice were housed at 20–23°C under a 12-h light/dark cycle. Male 5-week-old *p62* ^*KIKI*^ and *p62*-mRes mice were fed normal chow (NC) or a 60% high-fat/high-sucrose diet (HFD, Oriental Yeast, Tokyo, Japan) for 20 weeks. When the mice were 25 weeks of age, liver, skeletal muscle, epididymal adipose tissue (WAT), and blood samples were collected under isoflurane anesthesia. Blood samples were centrifuged at 1,500 × *g* for 10 min to collect serum. All the samples collected were stored at −80°C. The study was approved by the Animal Care and Use Committee of the University of Tsukuba, and was conducted in accordance with the Regulations on Animal Care and Use in Research; the Welfare and Management of Animals Act, and the Standards for Husbandry, Housing, and Pain Relief of Experimental Animals.

### Grip strength

The limb grip strength of the mice was measured at 17, 21, and 25 weeks of age using a grip strength meter for mice (MK-380K, Muromachi Kikai, Tokyo, Japan). The measurements were made by pulling the tail backward with the limbs touching the meter. The measurements were repeated five times and the mean values were recorded. All the measurements were performed by the same investigator.

### Histological analysis

The excised *tibialis anterior* (*TA*) muscles were snap-frozen in liquid nitrogen-cooled isopentane, cut into 10-μm-thick sections, and stained with hematoxylin and eosin (H&E) or for succinate dehydrogenase (SDH) activity. Immunofluorescence staining of muscle cryosection was performed after blocking with Blocking One Histo (nacalai tesque, Kyoto, Japan). The sections were incubated with primary antibodies diluted in PBS at 4°C overnight. After washing with PBS, sections were incubated with secondary antibodies labeled with Alexa 488 or Alexa 568 for 1 h at room temperature and mounted using Fluro KEEPER Antifade Reagent (nacalai tesque). The antibodies used for immunofluorescence staining are shown in Table 1. Liver sections were immersed in 4% paraformaldehyde, embedded in paraffin, cut into 5-μm-thick sections, and stained with H&E or Sirius Red. The liver pathologic grade was determined by a specialist using the SAF score (Bedossa et al., 2012) by assessing steatosis, activity (inflammation), and fibrosis. An all-in-one fluorescence microscope (BZ-X800, Keyence, Osaka, Japan) was used for imaging. Muscle fiber cross-sectional area (CSA), the percentage of type 1 fibers, and the percentages of the sections that were Sirius red-positive were calculated using BZ-H4C analysis software (Keyence).

**TABLE 1:** Antibodies used in this study.

Antibodies for immunofluorescent staining			
Antigen	Supplier	Catalog number	Dilution
Laminin 2α	Abcam	ab11576	1:500
Slow myosin heavy chain	Abcam	ab234431	1:200

Antibodies for immunoblotting			
p62/Sqstm1	Abcam	ab56416	1:1,000
p-mTOR (Ser 2,448)	CST	#2971	1:1,000
mTOR	CST	#2972	1:1,000
p-AKT (Ser 473)	CST	#4060	1:1,000
AKT	CST	#4691	1:1,000
GLUT4	SCB	sc-1608	1:1,000
β-actin	SCB	sc-47778	1:1,000

Abbreviations used in Table 1: CST (Cell Signaling Technology); SCB (Santa Cruz Biotechnology).

### Serum biochemistry

The serum concentrations of triglyceride (TG), non-esterified fatty acids (NEFA), high-density lipoprotein-cholesterol (HDL-C), low-density lipoprotein-cholesterol (LDL-C); and the activities of aspartate aminotransferase (AST), alanine aminotransferase (ALT), and lactate dehydrogenase (LDH) were measured by the Oriental Yeast using a Hitachi 7,180 Auto Analyzer. TG and NEFA were measured by enzymatic methods (L type Wako TG-M and NEFA HF, FUJIFILM, Tokyo, Japan). HDL-C and LDL-C were measured by direct methods (CHOLESTEST N LDL and HDL, S SEKISUI MEDICAL, Tokyo, Japan). AST and ALT activities were measured using the JSCC transferable method with L type Wako AST and ALT-J2 kits (FUJIFILM).

### Intraperitoneal glucose (ipGTT) and insulin (ipITT) tolerance testing

ipGTT was performed at 13 and 21 weeks of age, and ipITT at 14 and 22 weeks of age. For GTT, glucose solution (2 mg/g body mass) was administered intraperitoneally and the glucose concentration of tail vein blood was measured 0, 15, 30, 60, and 120 min later. For ITT, insulin (1 mU/g body mass) was administered intraperitoneally and the glucose concentration of tail vein blood was measured 0, 15, 30, 45, and 60 min later. The mice were fasted for 16 h before each test. A glucometer (SUGL-001, SHINYO GIKEN KOGYO Co., Ltd., Niigata, Japan) was used to measure the blood glucose concentrations.

### Real-time quantitative PCR (qPCR)

The expression levels of mRNAs were analyzed using qPCR, as described previously (Miura et al., 2021). RNA was extracted from *gastrocnemius* muscle or *liver* and cDNA was synthesized using a PrimeScript RT reagent kit (Takara Bio, Shiga, Japan). qPCR was performed using the cDNA and SYBR Green Master Mix (Thermo Fisher Scientific, MA, United States). The mRNA expression of target genes was normalized to that of the glyceraldehyde 3-phosphate dehydrogenase gene (*Gapdh*). The primers used for qPCR are shown in Table 2.

**TABLE 2:** Primers used for real-time quantitative PCR.

Gene	Forward primer (5'–3′)	Reverse primer (5'–3′)
*Pgc-1α*	TGCCCAGATCTTCCTG	TCTGTGAGAACCGCTA
*Tfam*	CTG​ATG​GGT​ATG​GAG​AAG​GAG​G	CCA​ACT​TCA​GCC​ATC​TGC​TCT​TC
*Ucp3*	TTT​CTG​CGT​CTG​GGA​GCT​T	GGC​CCT​CTT​CAG​TTG​CTC​AT
*Tnfα*	AAG​CCT​GTA​GCC​CAC​GTC​GTA	GGC​ACC​ACT​AGT​TGG​TTG​TCT​TG
*Il-1β*	TCC​AGG​ATG​AGG​ACA​TGA​GCA​C	GAA​CGT​CAC​ACA​CCA​GCA​GGT​TA
*Tgf-β1*	GTG​TGG​AGC​AAC​ATG​TGG​AAC​TCT​A	TTG​GTT​CAG​CCA​CTG​CCG​TA
*Col1a1*	GCA​CGA​GTC​ACA​CCG​GAA​CT	AAG​GGA​GCC​ACA​TCG​ATG​AT
*Gapdh*	AACGACCCCTTCATTGAC	TCC​ACG​ACA​TAC​TCA​GCA​C

### Immunoblot analysis

Tissue samples were homogenized in RIPA buffer (FUJIFILM) and the total protein concentration of each was measured using a BCA protein assay kit (Thermo Fisher Scientific). Protein lysate was mixed with Laemmli sample loading buffer (BioRad, CA, United States), and aliquots containing equal amounts of protein were separated by SDS-PAGE. The isolated proteins were transferred to PVDF membranes (BioRad), which were blocked using Blocking One (nacali tesque) and then incubated with primary antibodies. The target proteins were visualized using Chemi-Lumi One Super (nacali tesque). The antibodies used for immunoblotting are shown in Table 1. The target protein expression levels were quantified using Image J (NIH, MD, United States).

### Statistical analysis

SPSS statistics for Mac, version 26 (IBM Corp., Armonk, NY, United States) was used for the statistical analysis. Dates are expressed as mean ± standard error of the mean (SEM). Unpaired *t*-tests were used to compare two groups consuming the same diet. *p* < 0.05 was considered to represent statistical significance.

## Results

### p62 expression in muscle increases skeletal muscle mass, strength, and muscle fiber size, without affecting body mass

**FIGURE 2:**
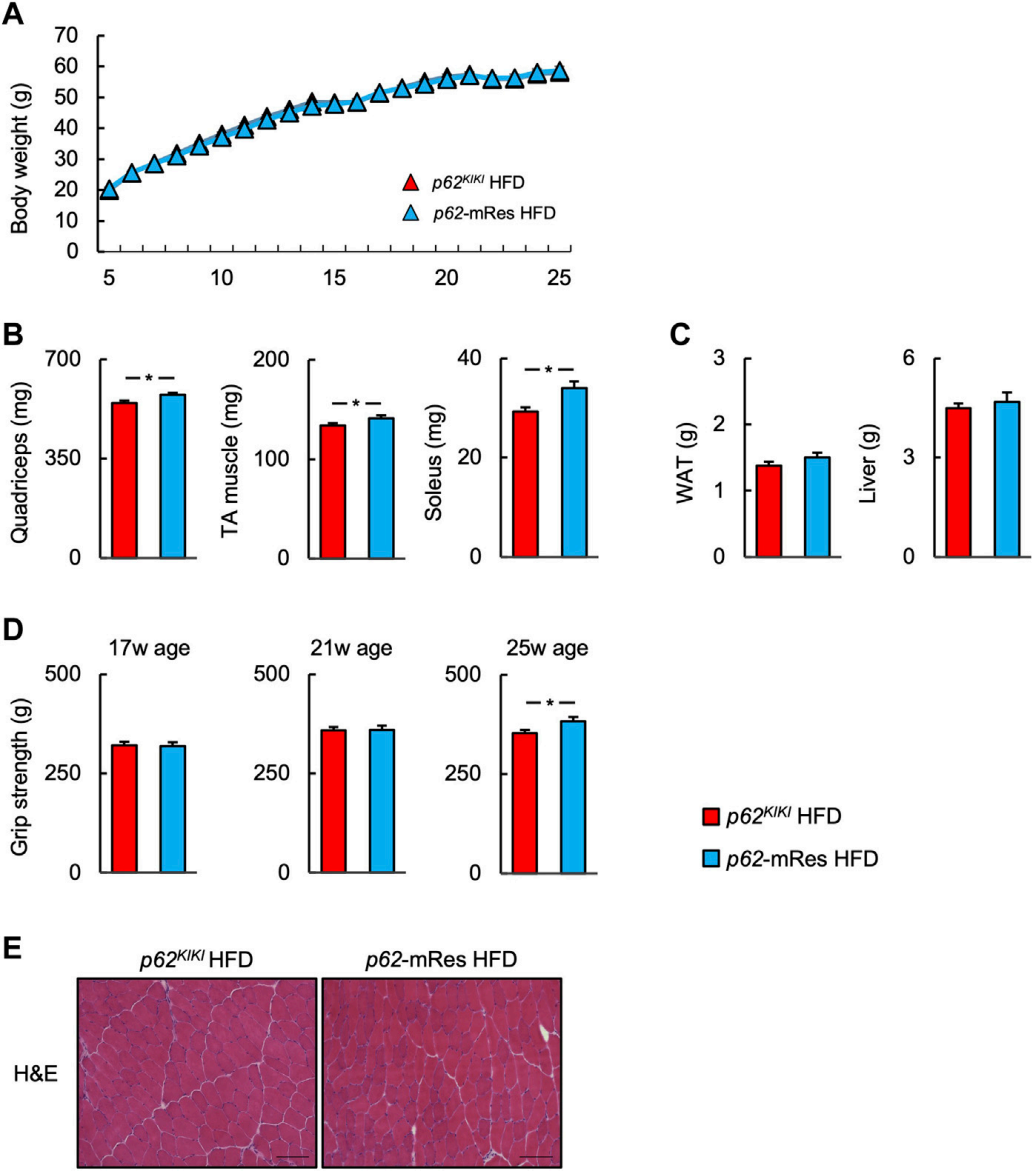
*p62* gene rescue in muscle does not affect body mass but increases skeletal muscle mass and grip strength. **(A)** Time course of the changes in body mass in *p62* ^*KIKI*^ and *p62*-mRes mice fed a high-fat diet (HFD, *p62* ^*KIKI*^; *n* = 13, *p62*-mRes; *n* = 14). **(B)** Masses of the *quadriceps*, *tibialis anterior* (TA), and *soleus* muscles of mice in each group at 25 weeks of age (*n* = 13–14/group). **(C)** Masses of the epididymal adipose tissue (WAT) and liver of mice in each group at 25 weeks of age (*n* = 13–14/group). **(D)** Grip strength at 17, 21, and 25 weeks of age (*n* = 13–14/group). **(E)** Representative hematoxylin and eosin (H&E)-stained sections of TA muscles from 25-week-old *p62* ^*KIKI*^ and *p62*-mRes mice fed an HFD. Scale bars represent 100 μm. Values are mean ± SEM. *p < 0.05 (unpaired *t*-test).

**FIGURE 3:**
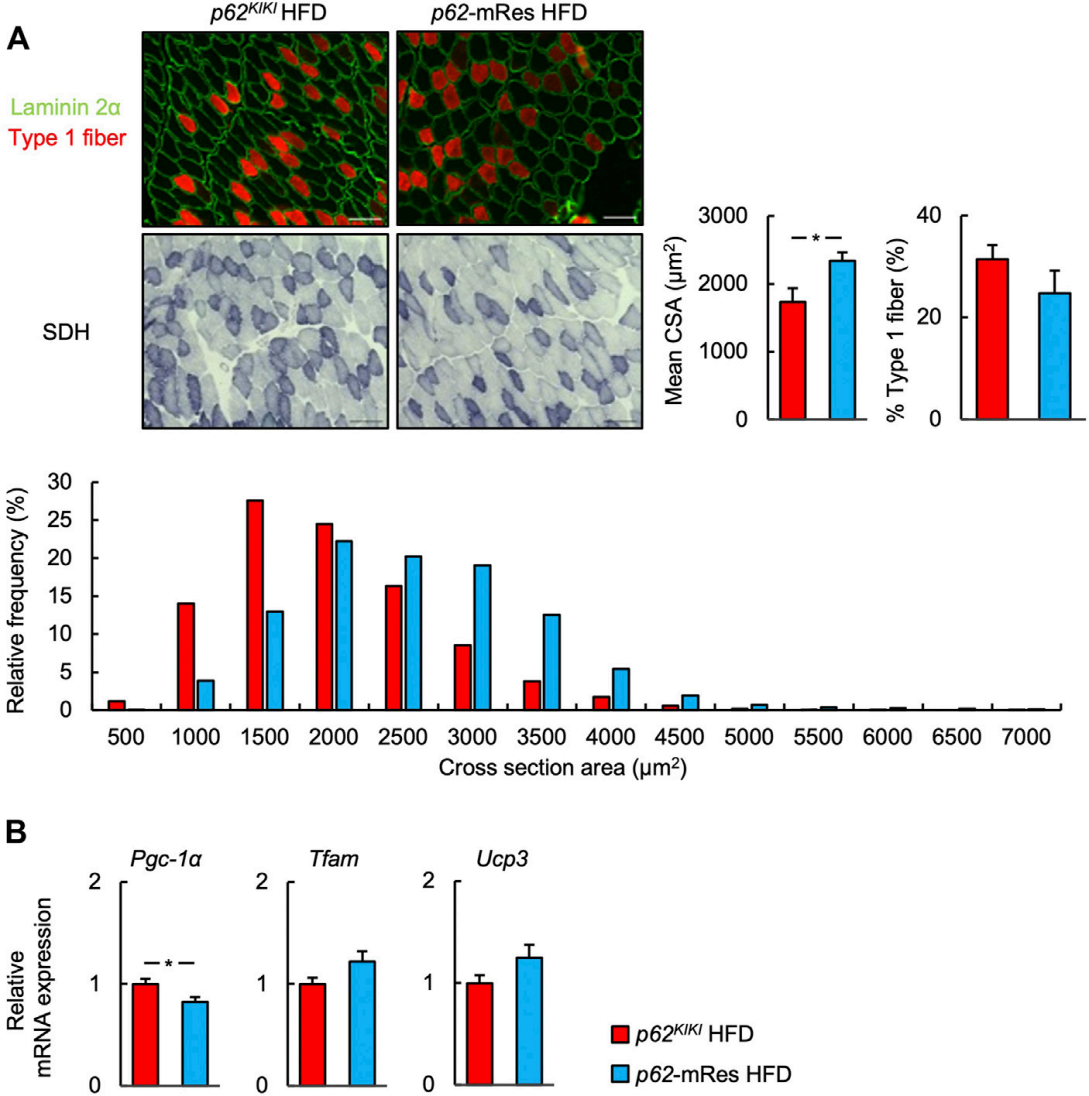
p62 in muscle increases muscle skeletal muscle fiber size. **(A)** Representative images of fluorescence immunostaining for laminin 2α (green) and slow myosin heavy chain (type 1 fibers, red), and succinate dehydrogenase (SDH)-stained sections of *tibialis anterior* muscles from 25-week-old *p62* ^*KIKI*^ and *p62*-mRes mice fed an HFD. Scale bars represent 100 μm. Skeletal muscle fiber cross-sectional area (CSA), percentage of type 1 fibers, and skeletal muscle fiber CSA distribution histograms for 25-week-old *p62* ^*KIKI*^ and *p62*-mRes mice fed an HFD (*n* = 4–5/group). **(B)** mRNA expression of mitochondria-related genes (*Pgc-1α*, *Tfam*, and *Ucp3*) by qPCR analysis (*n* = 8/group). Values are mean ± SEM. *p < 0.05 (unpaired *t*-test).

The *p62* gene rescue in muscle was confirmed by immunoblotting, and its expression in this tissue was comparable to that of wild-type mice (Figure 1). The *p62* ^*KIKI*^ and *p62*-mRes mice became obese with age (approximately 40 g) when consuming NC (Supplementary Figure S1A), and the consumption of an HFD accelerated the development of the obesity (Figure 2A), but muscle p62 did not affect their body mass. The masses of the *quadriceps*, *TA*, and *soleus* muscles tended to be higher in the *p62*-mRes mice, and the masses of all the muscles were higher in *p62*-mRes fed an HFD than in *p62* ^*KIKI*^ mice that consumed the same diet (Figure 2B, Supplementary Figure S1B). The masses of the epididymal adipose tissue (WAT) and liver did not change by genotype (Figure 2C, Supplementary Figure S1C). The grip strengths of *p62*-mRes mice fed NC at 17 and 21 weeks of age were higher than in *p62* ^*KIKI*^ mice consuming NC (Supplementary Figure S1D), and at 25 weeks of age, it was higher in *p62*-mRes mice consuming an HFD than in *p62* ^*KIKI*^ mice consuming the same diet (Figure 2D). No morphological differences were identified in the skeletal muscle of mice that did or did not locally express p62, and none of the mice showed abnormal skeletal muscle morphology (Figure 2E, Supplementary Figure S1E). Similar to the skeletal muscle mass and strength, the CSAs of the muscle fibers were also higher in HFD-fed *p62*-mRes mice, and a comparison of the distributions of the CSAs of the fibers showed that the HFD-fed *p62*-mRes mice had many large fibers (Figure 3A). The percentage of type 1 fibers was not affected by muscle p62 expression (Figure 3A, Supplementary Figure S2A). There were no differences in SDH staining between mice that consumed the different diets or those of differing genotype (Figure 3A, Supplementary Figure S2A), which suggests that p62 did not affect the mitochondrial content of the skeletal muscle of the mice. qPCR analysis showed that the expression of *Pgc-1a*, which is involved in mitochondrial biosynthesis, was lower in *p62*-mRes mice (Figure 3B), whereas other markers of mitochondrial biosynthesis and function (*Tflam* and *Ucp3*) were similar in the two mouse strains (Figure 3B). In the NC-fed mice, only *Tfam* expression differed (Supplementary Figure S2B). These results indicate that p62 in muscle does not affect the obesity associated with *p62* gene deletion, but increases skeletal muscle mass, strength, and fiber size.

### p62 expression in muscle reduces the serum concentrations of NEFA and LDL-C

**FIGURE 4:**
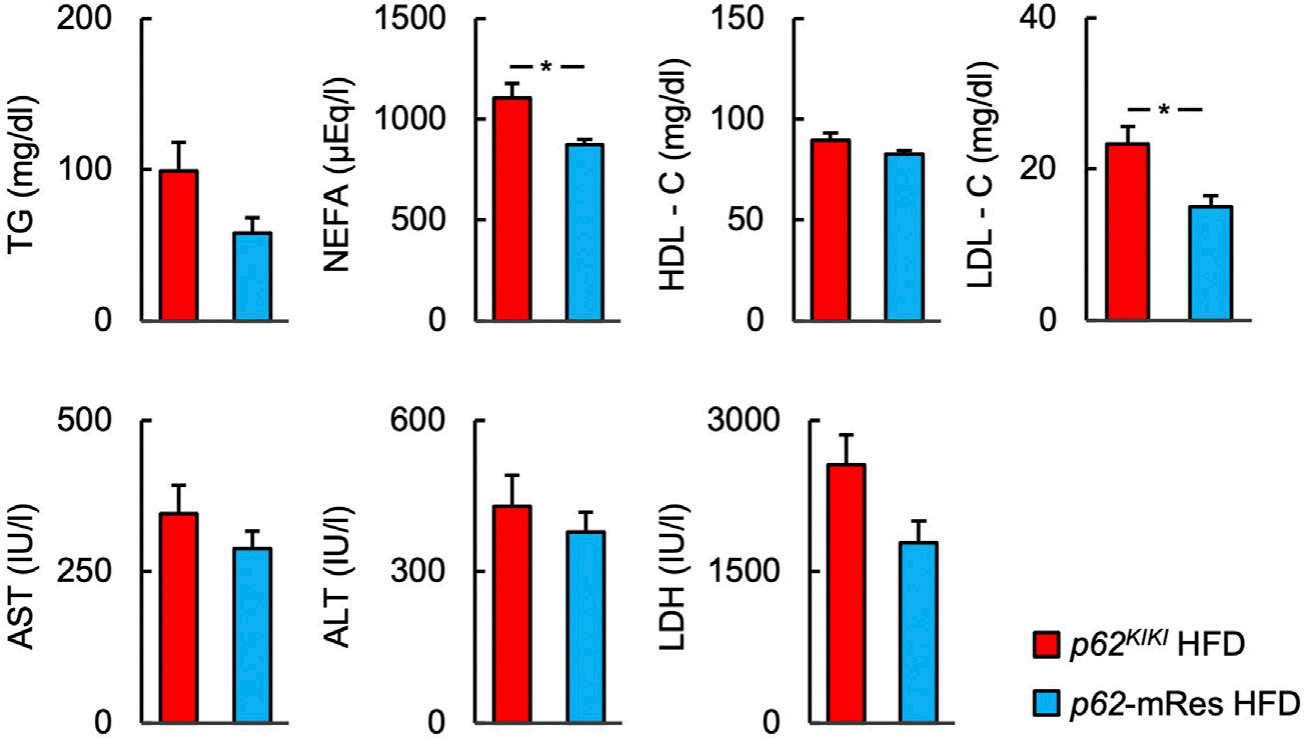
p62 in muscle reduces the serum concentrations of NEFA and LDL-C. Serum biochemistry [the triglyceride (TG), non-esterified fatty acids (NEFA), high-density lipoprotein-cholesterol (HDL-C), and low-density lipoprotein-cholesterol (LDL-C) concentrations; and the aspartate aminotransferase (AST), alanine aminotransferase (ALT), and lactate dehydrogenase (LDH) activities] in the fed state (*n* = 6/group). Values are mean ± SEM. *p < 0.05 (unpaired *t*-test).

Serum biochemical parameters were measured under fed conditions (Figure 4, Supplementary Figure S3A). Although the serum TG concentration was not affected by p62 expression in muscle, the serum NEFA concentration of *p62*-mRes mice consuming an HFD was significantly lower than that of *p62* ^*KIKI*^ mice consuming the same diet. The serum HDL-C concentration was not significant. The serum LDL-C concentration was lower in *p62*-mRes mice consuming an HFD than in *p62* ^*KIKI*^ mice consuming same diet. The circulating ALT, AST, and LDH activities were not affected by p62 expression in muscle.

### p62 in muscle ameliorates obesity-induced glucose intolerance and insulin resistance

**FIGURE 5:**
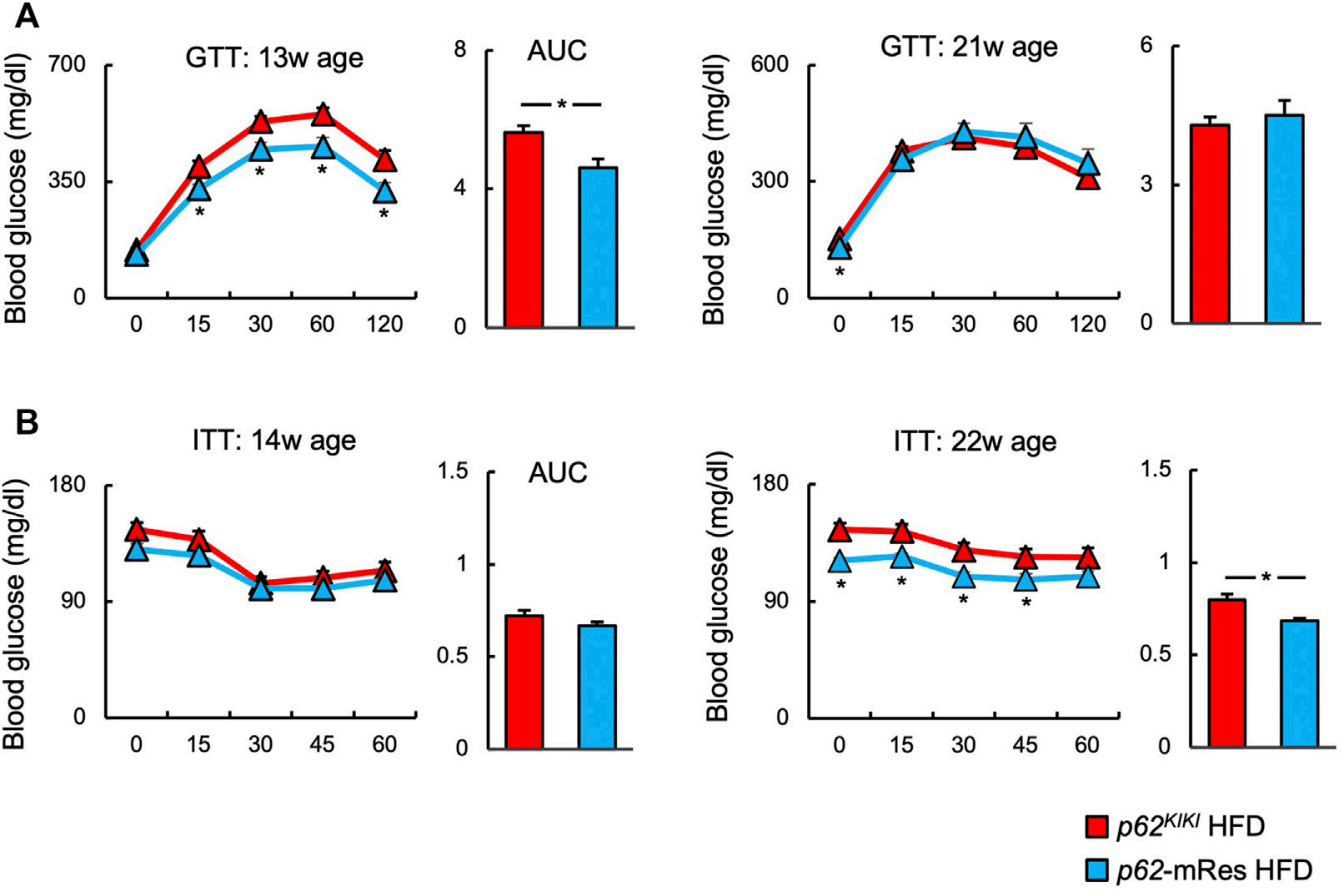
p62 in muscle ameliorates obesity-associated glucose intolerance and insulin resistance. **(A)** Changes in blood glucose concentration and area under the curve (AUC) during intraperitoneal glucose tolerance testing (ipGTT) at 13 and 21 weeks of age in HFD-fed (*n* = 8–14/group) mice. **(B)** Changes in blood glucose concentration and area under the curve during intraperitoneal insulin tolerance testing (ipITT) at 14 and 22 weeks of age in HFD-fed (*n* = 10–14/group) mice. Values are mean ± SEM. *p < 0.05 (unpaired *t*-test).

The glucose tolerance and insulin resistance of mice with or without muscle p62 expression were evaluated using ipGTT and ipITT. In both the NC and HFD-fed groups, ipGTT at 13 weeks of age was characterized by lower blood glucose concentrations following glucose administration and a smaller area under the glucose curve in those with *p62* gene rescue in muscle (Figure 5A, Supplementary Figure S3B). However, only the NC group showed lower post-glucose load blood glucose concentration, and area under the curve on ipGTT at 21 weeks of age (Supplementary Figure S3B). These findings imply that p62 in muscle improves obesity-associated glucose intolerance. At 14 and 22 weeks of age, there were no clear differences on ipITT in the NC group (Supplementary Figure S3B), but at 22 weeks of age, HFD-fed *p62*-mRes mice showed lower fasting and post-insulin blood glucose concentrations and area under the curve (Figure 5B). Thus, p62 in muscle protects against severe obesity-induced insulin resistance in mice.

### p62 in muscle is involved in the activation of mTOR and helps determine GLUT4 expression in skeletal muscle

**FIGURE 6:**
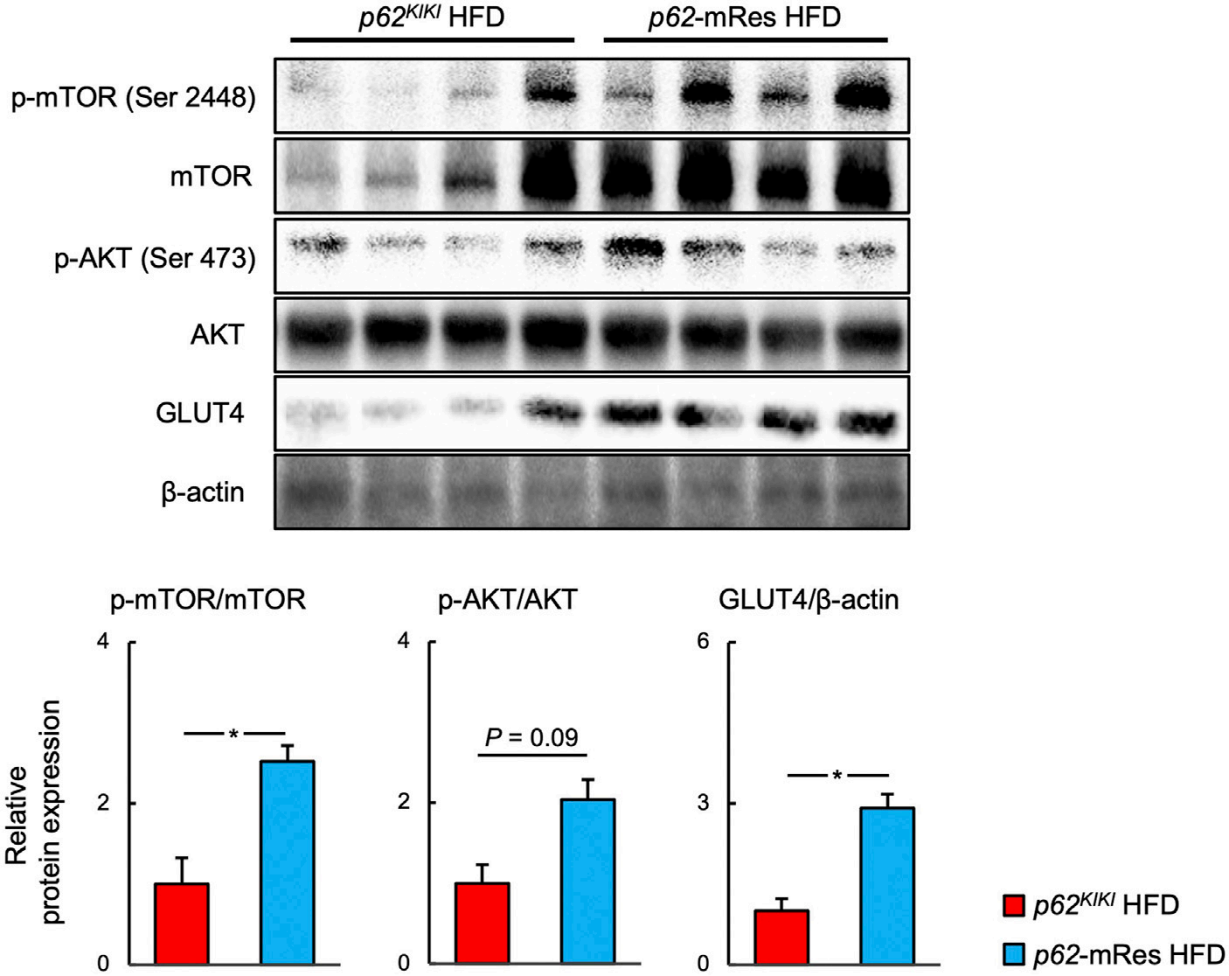
p62 in muscle activates mTOR and increases the expression of GLUT 4. Representative immunoblots of *quadriceps* muscle for the phosphorylation of mTOR (p-mTOR, Ser 2,448), total mTOR, phosphorylation of AKT (p-AKT, Ser 473), total AKT, GLUT4, and β-actin in *p62* ^*KIKI*^ and *p62*-mRes mice fed an HFD. Quantification of protein levels. p-mTOR and p-AKT levels were normalized to those of total mTOR or total AKT. GLUT4 levels were normalized to those of β-actin (*n* = 5–6/group). Values are mean ± SEM. *p < 0.05 (unpaired *t*-test).

Immunoblotting analyses were performed to investigate the molecular mechanism by which p62 in muscle increases muscle mass and ameliorates insulin resistance in HFD-fed mice (Figure 6). The phosphorylation (p-mTOR ser 2,448) and total protein expression of mTOR were higher in the muscle of *p62*-mRes mice than in *p62* ^*KIKI*^ mice, implying that mTOR activation is increased by p62. The phosphorylation of AKT (p-AKT Ser 473) also tended to be higher. Furthermore, the expression of the insulin-stimulated glucose transporter GLUT4 in skeletal muscle was higher in HFD-fed *p62*-mRes. Thus, p62 in muscle may promote skeletal muscle protein synthesis by activating mTOR and ameliorate insulin resistance via the AKT-GLUT4 pathway.

### p62 in muscle retards the progression of NASH

**FIGURE 7:**
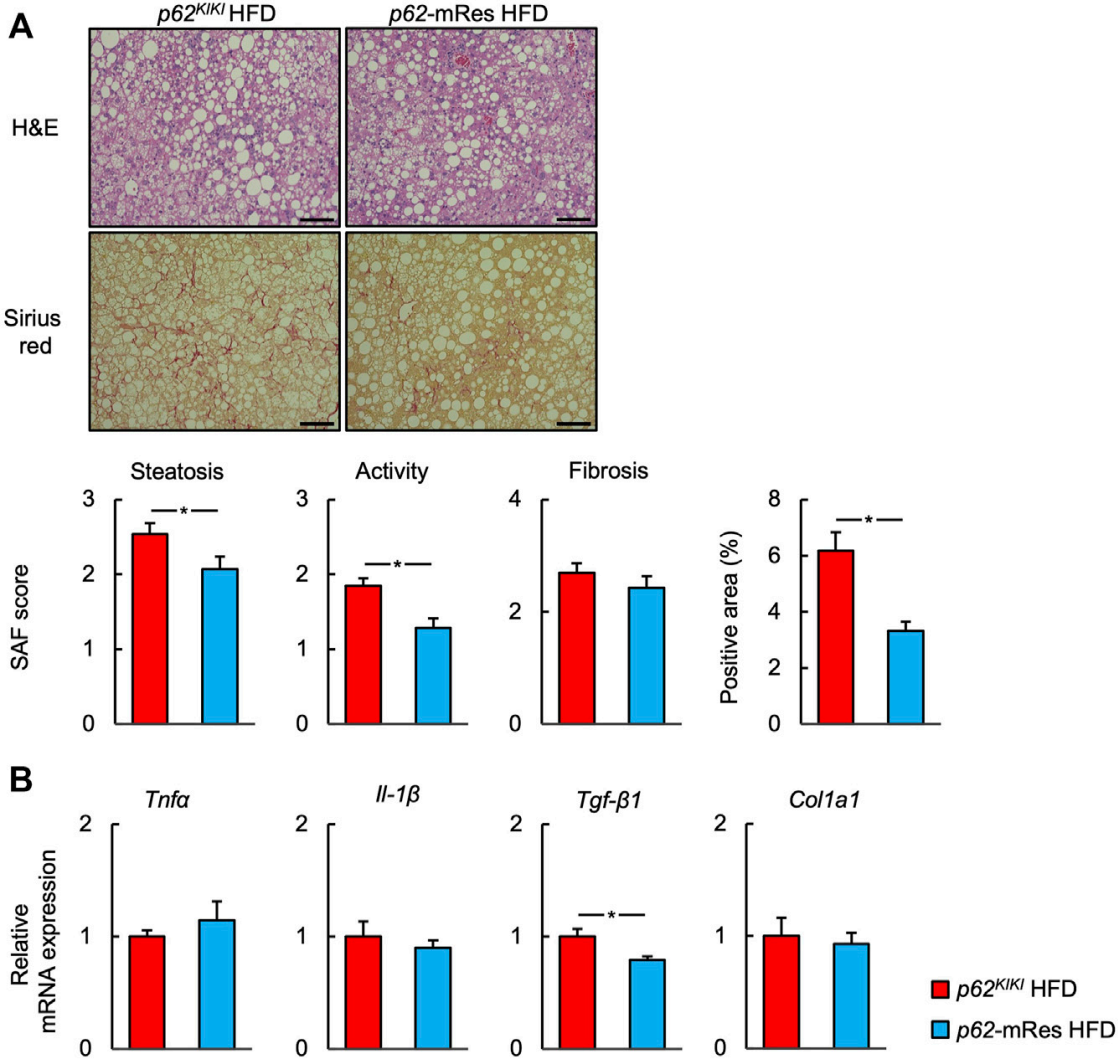
p62 expression in muscle retards the progression of NASH. **(A)** Representative hematoxylin and eosin (H&E) and Sirius red-stained sections of liver from 25-week-old *p62* ^*KI/KI*^ and *p62*-mRes mice fed an HFD. Scale bars represent 100 μm. The steatosis, activity, and fibrosis (SAF) score and area positive for Sirius red staining (*n* = 13–14/group). **(B)** mRNA expression of proinflammatory cytokines (*Tnfα* and *Il-1β*) and fibrosis-related genes (*Tgf-β1*, and *Col1a1*) (*n* = 8/group). Values are mean ± SEM. *p < 0.05 (unpaired *t*-test).

*p62*-KO mice develop NASH, featuring hepatic inflammation and fibrosis, when fed an HFD (Duran et al., 2016; Miura et al., 2021). Because skeletal muscle insulin resistance is associated with the development and progression of NASH (Bhanji et al., 2017), we next investigated whether the maintenance of skeletal muscle function by p62 would affect the liver pathology of the mice. Histological analysis using the SAF score showed that HFD-feeding caused NASH. Interestingly, the hepatic steatosis and inflammation of *p62*-mRes mice were less marked compared with those of *p62* ^*KIKI*^ mice (Figure 7A). There was no significant difference in the fibrosis score, but there tended to be less fibrosis in *p62*-mRes mice and there was a significantly smaller area of Sirius red staining (Figure 7A). Similar to the findings of previous studies, the NC-fed mice did not develop NASH (Supplementary Figure S4). qPCR analysis showed that the expression of *Tgf-β1*, which promotes liver fibrosis, was lower in *p62*-mRes mice, but the expression of other markers of inflammation (*Tnfa* and *Il-1β*) and fibrosis (*Col1a1*) were unaffected (Figure 7B). Thus, there were no differences in the expression of genes encoding pro-inflammatory cytokines; the cytokine gene that was expressed at changes in *p62*-mRes mice may be considered to be anti-inflammatory and pro-fibrotic. These findings imply that p62 in muscle retards the progression of NASH via a muscle-liver axis.

## Discussion

In the present study, we have used our newly generated muscle-specific *p62* gene rescue mice (*p62*-mRes) to demonstrate that p62 in muscle 1) increases muscle mass and strength during HFD-feeding, 2) improves glucose tolerance and insulin resistance without affecting body mass, 3) activates mTOR signaling and increases GLUT4 expression, and 4) retards the progression of NASH, a common of feature of the metabolic syndrome.

The insulin resistance associated with overweight/obesity is a risk factor for many diseases (Kodama et al., 2014; Younossi, 2019), and it is important to understand the mechanisms whereby organisms may protect themselves against this. *p62*-KO mice show hyperphagia-induced obesity secondary to abnormal leptin signaling. They exhibit a phenotype similar to metabolic syndrome in humans, which includes insulin resistance, glucose intolerance, and NAFL; and HFD-feeding exacerbates these defects (Okada et al., 2009; Harada et al., 2013; Duran et al., 2016; Miura et al., 2021). However, it remains to be determined whether the phenotype of the *p62*-KO mice is the result of the deletion of the *p62* gene or is simply obesity-related. Additionally, the skeletal muscle is an important target of insulin, given that represents the largest glucose store in the body, but the role of p62 in muscle remains to be fully characterized. To address these deficiencies, we generated *p62*-mRes mice, which express p62 only in muscle, and were derived from *p62*-knock-in mice (*p62* ^*KIKI*^, Figure 1). Both the *p62* ^*KIKI*^ and *p62*-mRes mice developed severe obesity when fed an HFD, but p62 expression in muscle did not affect the body mass of the mice (Figure 2A). The masses of the epididymal adipose tissue (WAT) depots were also not affected by genotype (Figure 2C, Supplementary Figure S1C), despite the HFD-induced increase in body mass. These results suggest that the HFD-fed *p62/Sqstm1* knock-out mice may have a lipodystrophic phenotype. This implies that p62 in muscle does not affect energy expenditure or prevent the development of obesity.

*p62* gene rescue in muscle increased the limb skeletal muscle mass, strength, and fiber size of the HFD-fed mice (Figures 2B,D, Figure 3A). We also identified the activation of mTOR in the skeletal muscle of the muscle p62-expressing mice (Figure 6), which may be responsible for this phenotype. mTOR is a key positive regulator of muscle protein synthesis (Bodine et al., 2001; Glass, 2005). The relationship between p62 and mTOR in skeletal muscle is unclear, but it has been reported that p62 increases mTOR activity in other tissues and cells (Duran et al., 2011; Katsuragi et al., 2015). Although it has previously been shown that overweight is associated with higher skeletal muscle mass (Newman et al., 2003), in the present study, the muscle mass and muscle fiber size were greater in HFD-fed *p62*-mRes mice, but not in HFD-fed *p62* ^*KIKI*^ mice (Figures 2B,D, Figure 3A). These results suggest that *p62* gene deletion causes abnormal mTOR signaling and attenuates muscle protein synthesis in *p62* ^*KIKI*^, but *p62* gene rescue in muscle restores mTOR activation, which may result in muscle hypertrophy. Thus, p62 may be a positive regulator of protein synthesis in skeletal muscle.

Muscle-specific *p62* expression reduced the fasting blood glucose concentration of the mice and improved their glucose tolerance and insulin resistance when consuming an HFD (Figure 5). Increases in circulating NEFA concentrations cause insulin resistance (Kahn et al., 2006), and p62 expression in muscle may at least in part ameliorate insulin resistance by reducing serum NEFA and LDL-C concentrations (Figure 4). It has been reported that p62 is involved in mitochondrial function in brown adipose tissue, and that *p62* deficiency causes decreases in mitochondrial oxygen consumption capacity and thermogenesis in brown adipose tissue (Muller et al., 2013). However, the relationship between p62 and mitochondrial respiratory capacity in muscle has not been characterized. In the present study, the gene expression of *Pgc1a* in muscle was lower in HFD-fed *p62*-mRes mice, which was not consistent with the expression of *Tfam* and *Ucp3*. Moreover, no difference in SDH staining was observed, so we have no evidence that muscle mitochondrial content or activity is altered by muscle *p62* expression. However, in a previous study, *p62* deficiency was associated with lower expression of Pgc-1α in BAT (Muller et al., 2013). Thus, when taken together with the lower serum NEFA and LDL-C concentrations in *p62*-mRes mice, the results of the present study suggest that p62 may promote mitochondrial fatty acid uptake and β-oxidation in skeletal muscle by different mechanisms to its expression in BAT. Thus, it is possible that p62 may contribute to the amelioration of insulin resistance by enhancing lipid metabolism in skeletal muscle. In addition, the translocation of GLUT4 to the cell membrane in response to the phosphorylation of AKT is a critical step in insulin-stimulated glucose uptake in skeletal muscle. In HFD-fed *p62*-mRes mice, the phosphorylation of AKT (Ser 473) tended to be higher and GLUT4 protein expression was higher than that of *p62* ^*KIKI*^ mice (Figure 6). It has been reported that the phosphorylation of AKT (Ser 473) is increased via the activation of NF-E2-related factor 2 (Nrf2) in mice overexpressing *p62* in hepatocytes, resulting in a reduction in serum glucose concentration (He et al., 2020). Because there were no differences in the body mass of the *p62*-mRes and *p62* ^*KIKI*^ mice, it is possible that *p62* gene rescue in muscle ameliorates the glucose intolerance and insulin resistance of the mice through an increase in GLUT4 expression.

AKT is a regulator of mTOR, and its phosphorylation induces mTOR activation via the inhibition of TSC1/2 (Bodine et al., 2001). The most likely explanation is that p62 is involved in phosphorylation of AKT, and thereby both promotes glucose uptake in skeletal muscle via GLUT4, and activates mTOR, contributing to the increases in skeletal muscle mass and strength. However, although amino acid stimulation induces mTOR activation independently of AKT signaling (Yoon, 2017), a previous study showed that p62 increases the sensitivity of mTOR to amino acids (Duran et al., 2011; Sugiyama et al., 2017). p62 interacts with raptor, a component of mTOR complex 1 (mTORC1) in the presence of amino acids and plays an important role in the recruitment of mTORC1 to lysosomes, a critical step in mTORC1 activation (Duran et al., 2011). Furthermore, *p62*-deficient MEF cells show lower amino acid-induced mTOR activation than wild-type cells, but the deletion of *p62* does not affect AKT-mediated mTOR activation in response to insulin, suggesting that p62 promotes mTOR activation independently of AKT. Because there is a link between skeletal muscle mass and insulin resistance, a resistance training-induced increase in skeletal muscle mass ameliorates insulin resistance (Bucci et al., 2016). Therefore, the AKT-independent activation of mTOR by p62 and the resulting increase in skeletal muscle mass might at least in part provide the mechanism by which p62 in muscle ameliorates insulin resistance independent of the AKT-GLUT4 pathway. The association of p62 with AKT and GLUT4, or mTOR, and the detailed molecular mechanisms whereby p62 induces an increase in skeletal muscle mass and an amelioration of insulin resistance require further study.

A number of extra-hepatic factors are involved in the development and progression of NASH (Tilg and Moschen., 2010), and it has been shown that skeletal muscle loss (sarcopenia) and insulin resistance in skeletal muscle promote the progression of NASH via a muscle-liver axis (Bhanji et al., 2017). In the present study, HFD-feeding induced NASH, including hepatic inflammation and fibrosis, in *p62* ^*KIKI*^ mice, whereas *p62* gene rescue in muscle retarded the progression of NASH, including hepatic steatosis, inflammation, and fibrosis, without affecting body mass (Figure 7A). Additionally, the hepatic mRNA expression of *Tgf-β1*, a profibrotic factor, was low in *p62*-mRes mice (Figure 7B). The hyperglycemia and hyperinsulinemia associated with insulin resistance activate hepatic stellate cells (HSCs) and promote hepatic fibrosis (Svegliati-Baroni et al., 1999; Sugimoto et al., 2005), suggesting that the improvement in glucose metabolism induced by p62 in muscle may contribute to the retardation of NASH progression via a muscle-liver axis. There is no established means of preventing or treating NAFLD/NASH, other than diet and exercise therapy. Exercise ameliorates the hepatic steatosis and fibrosis independently of weight loss (Oh et al., 2021), and in addition, regular exercise activates p62 in skeletal muscle (Yamada et al., 2019). The results of the present study suggest that muscle p62 may play a role in the amelioration of insulin resistance and NAFLD/NASH induced by regular exercise, and imply that further studies should be performed regarding the relationship between exercise training and p62.

There were several limitations to the present study. First, because wild-type mice were not used in the present study, we could not directly evaluate the extent to which muscle-specific *p62* gene expression restores the muscle atrophy, insulin resistance, and NASH induced by *p62* deficiency. Therefore, in the future, more detailed analysis, including of wild-type mice and muscle-specific *p62* knock-out mice will be necessary. Second, we did not conduct a detailed analysis of body composition. Therefore, it would be important to analyze the changes in visceral fat mass induced by p62 expression in muscle, which influences the development of insulin resistance and NASH, by means of magnetic resonance imaging and other methods. Third, although muscle p62 expression reduced the serum NEFA concentration, the underlying mechanism has not been fully established. Because high circulating serum NEFA concentration is a risk factor for insulin resistance, an investigation of the direct effects of muscle p62 expression on fatty acid uptake and mitochondrial respiratory capacity in skeletal muscle should also be performed in the future. Fourth, although adipose tissue, along with skeletal muscle and liver, is involved in the pathogenesis of insulin resistance, there has been a lack of analysis of the relationship between insulin resistance in adipose tissue and parameters such as the fasting circulating insulin and NEFA concentrations. The muscle-fat and muscle-liver-fat axes represent important avenues of research to improve understanding of the mechanism of whole-body insulin resistance. Future studies should analyze the mechanism of cross-talk between organs mediated by p62 that are involved in insulin resistance.

In summary, we have shown that p62 expression in muscle increases muscle mass and strength and ameliorates glucose intolerance and insulin resistance, all of which likely contribute to a retardation of the HFD-induced progression of NASH in a transgenic mouse model. The p62-associated improvements in skeletal muscle mass and function may be mediated through greater activation of mTOR and an increase in GLUT4 in muscle. Thus, muscle p62 may represent a molecular target for the management of skeletal muscle mass and function in obesity.

## Data Availability

The raw data supporting the conclusions of this article will be made available by the authors, without undue reservation.

## Supplementary material

Supplementary Figure 1 Changes in body composition in the NC-fed mice. **(A)** Time course of the changes in body mass in *p62* ^KIKI^ and *p62*-mRes mice fed normal chow (NC, *p62* ^KIKI^; *n* = 12, *p62*-mRes; *n* = 8). **(B)** Masses of the quadriceps, *tibialis anterior* (TA), and *soleus* muscles of mice in each group at 25 weeks of age (*n* = 8–12/group). **(C)** Masses of the epididymal adipose tissue (WAT) and liver of mice in each group at 25 weeks of age (*n* = 8–12/group). **(D)** Grip strength at 17, 21, and 25 weeks of age (*n* = 7–12/group). **(E)** Representative hematoxylin and eosin (H&E)-stained sections of TA muscles from 25-week-old *p62* ^KIKI^ and *p62*-mRes mice fed NC. Scale bars represent 100 μm. Values are mean ± SEM. * *p* < 0.05 (unpaired t-test).

Supplementary Figure 2 Analysis of muscle fiber size and type and mitochondria-related parameters in the NC-fed mice. **(A)** Representative images of fluorescence immunostaining for laminin 2α (green) and slow myosin heavy chain (type 1 fibers, red), and succinate dehydrogenase (SDH)-stained sections of *tibialis* anterior muscles from 25-week-old *p62* ^KIKI^ and *p62*-mRes mice fed NC. Scale bars represent 100 μm. Skeletal muscle fiber cross-sectional area (CSA), percentage of type 1 fibers, and skeletal muscle fiber CSA distribution histograms for 25-week-old *p62* ^KIKI^ and *p62*-mRes mice fed NC (*n* = 4/group). **(B)** mRNA expression of mitochondria-related genes (*Pgc-1α, Tfam, and Ucp3*) by qPCR analysis (*n* = 8/group). Values are mean ± SEM. * *p* < 0.05 (unpaired t-test).

Supplementary Figure 3 Evaluation of blood biochemistry, glucose tolerance and insulin resistance in the NC-fed mice. **(A)** Serum biochemistry (the triglyceride [TG], non-esterified fatty acids [NEFA], high-density lipoprotein-cholesterol [HDL-C], and low-density lipoprotein-cholesterol [LDL-C] concentrations; and the aspartate aminotransferase [AST], alanine aminotransferase [ALT], and lactate dehydrogenase [LDH] activities) in the fed state (*n* = 6/group). **(B)** Changes in blood glucose concentration and area under the curve (AUC) during intraperitoneal glucose tolerance testing (ipGTT) at 13 and 21°weeks of age in NC-fed (*n* = 7–12/group) mice. Changes in blood glucose concentration and area under the curve during intraperitoneal insulin tolerance testing (ipITT) at 14 and 22°weeks of age in NC-fed (*n* = 7–12/group) mice. Values are mean ± SEM. * *p* < 0.05 (unpaired t-test)

Supplementary Figure 4 Evaluation of liver lesions in the NC-fed mice. **(A)** Representative hematoxylin and eosin (H&E) and Sirius red-stained sections of liver from 25-week-old *p62* ^KI/KI^ and *p62*-mRes mice fed NC. Scale bars represent 100 μm. **(B)** mRNA expression of proinflammatory cytokines (Tnfα and Il-1β) and fibrosis-related genes (*Tgf-β1, and Col1a1*) (*n* = 8/group). Values are mean ± SEM. * *p* < 0.05 (unpaired t-test).

## Abbreviations

AKT, Protein kinase B; ALT, alanine aminotransferase; AST, aspartate aminotransferase; Col1a1, collagen type 1 alpha; GLUT4, glucose transporter type 4; H&E, hematoxylin and eosin; HDL-C, high-density lipoprotein cholesterol; Il-1β, interleukin 1 beta; LDH, lactate dehydrogenase; LDL-C, low-density lipoprotein cholesterol; mTOR, mechanistic target of rapamycin; NEFA, non-esterified fatty acid; Pgc-1α, peroxisome proliferator-activated receptor gamma coactivator 1 alpha; SDH, succinate dehydrogenase; Tfam, mitochondrial transcription factor A; TG, triglyceride; Tgf-β1, transforming growth factor beta 1; Tnfα, tumor necrosis factor alpha; Ucp3, uncoupling protein 3.
