# Supplementary figures and images for: p62/Sqstm1 rescue in muscle retards the progression of steatohepatitis in p62/Sqstm1-null mice fed a high-fat diet
Source: Front Physiol. 2022 Nov 1;13:993995. doi: 10.3389/fphys.2022.993995 (PMC9692207; doi:10.3389/fphys.2022.993995)

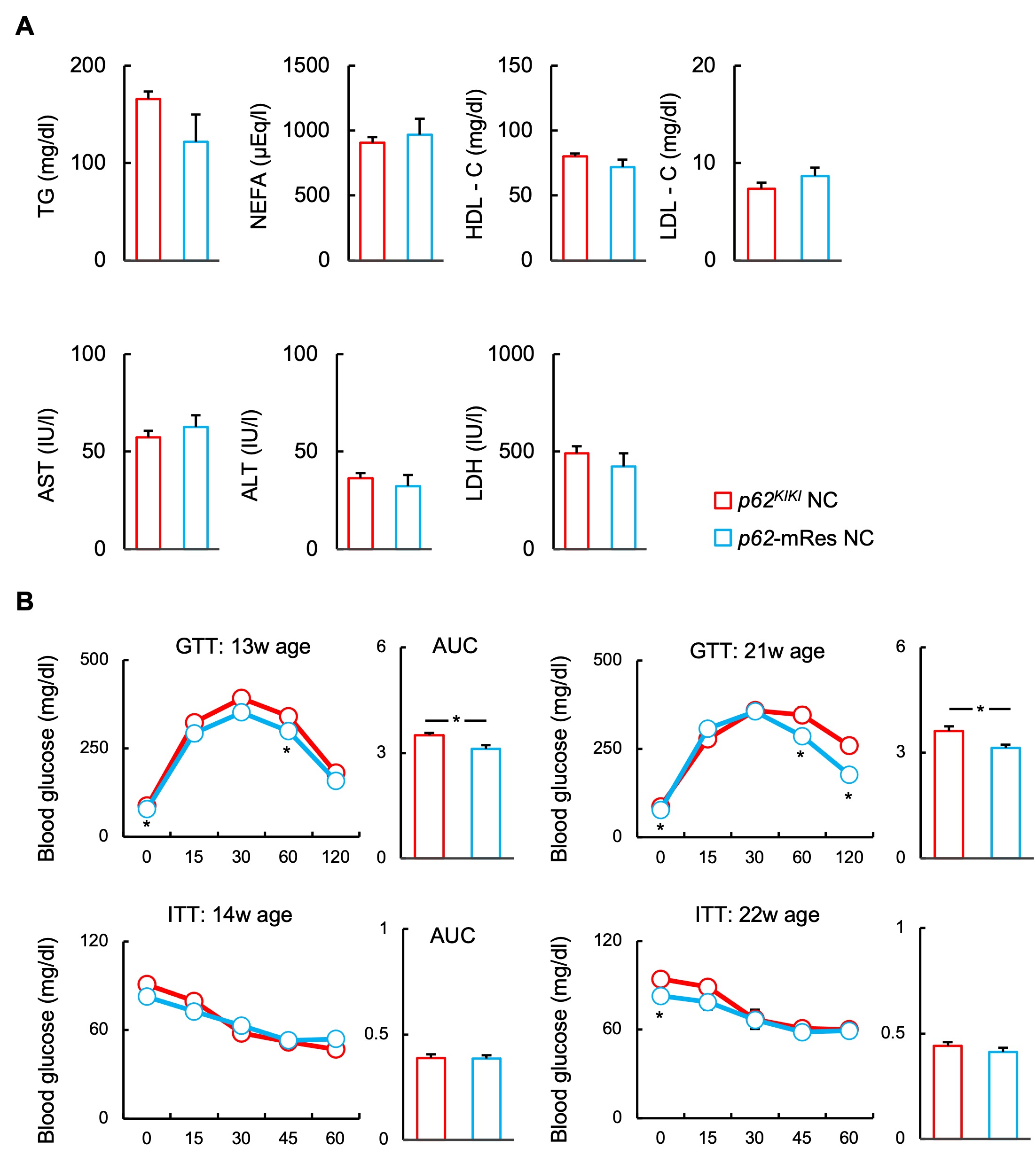

Supplement: Supplementary file 1 [file Image3.JPEG]

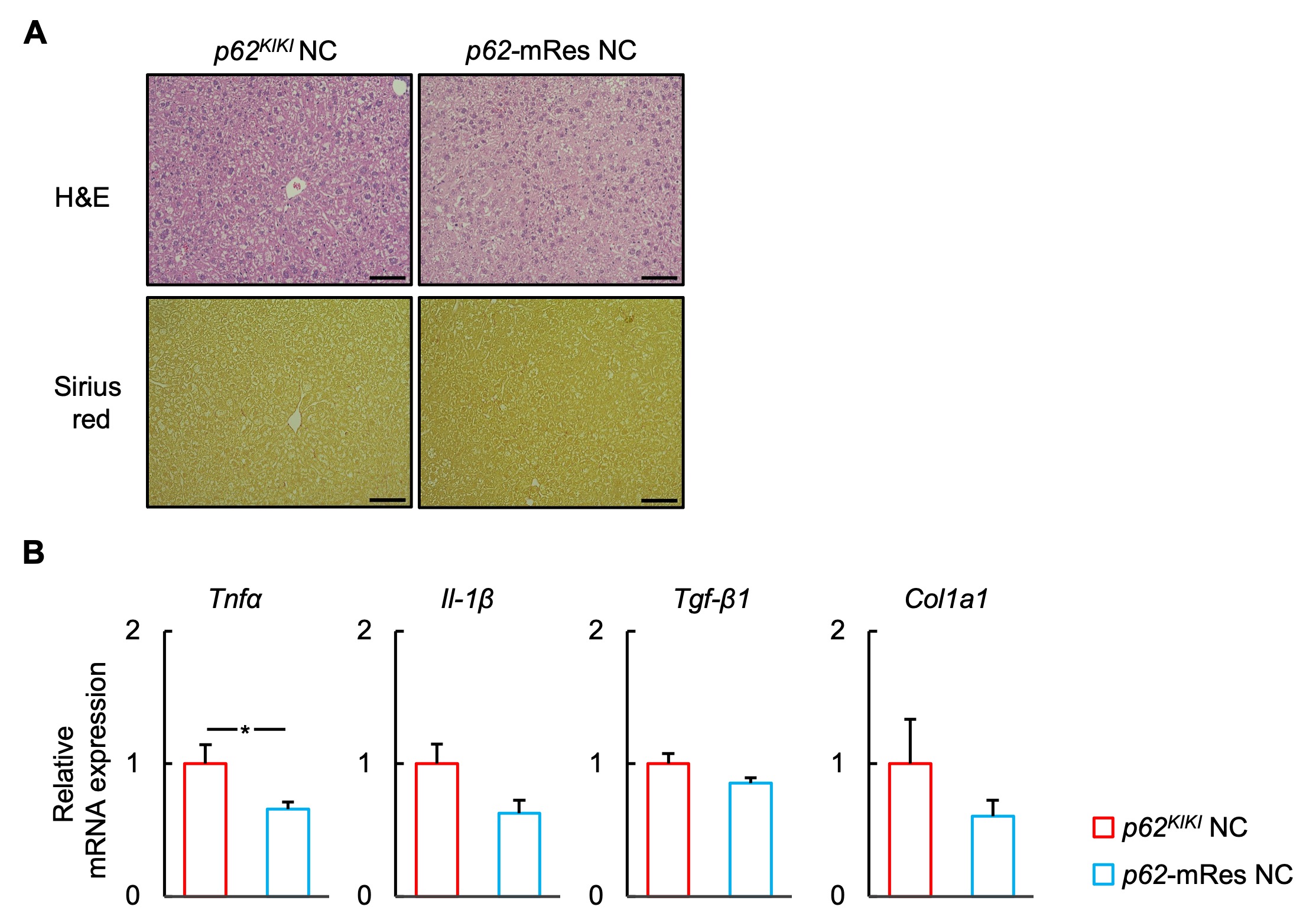

Supplement: Supplementary file 2 [file Image4.JPEG]

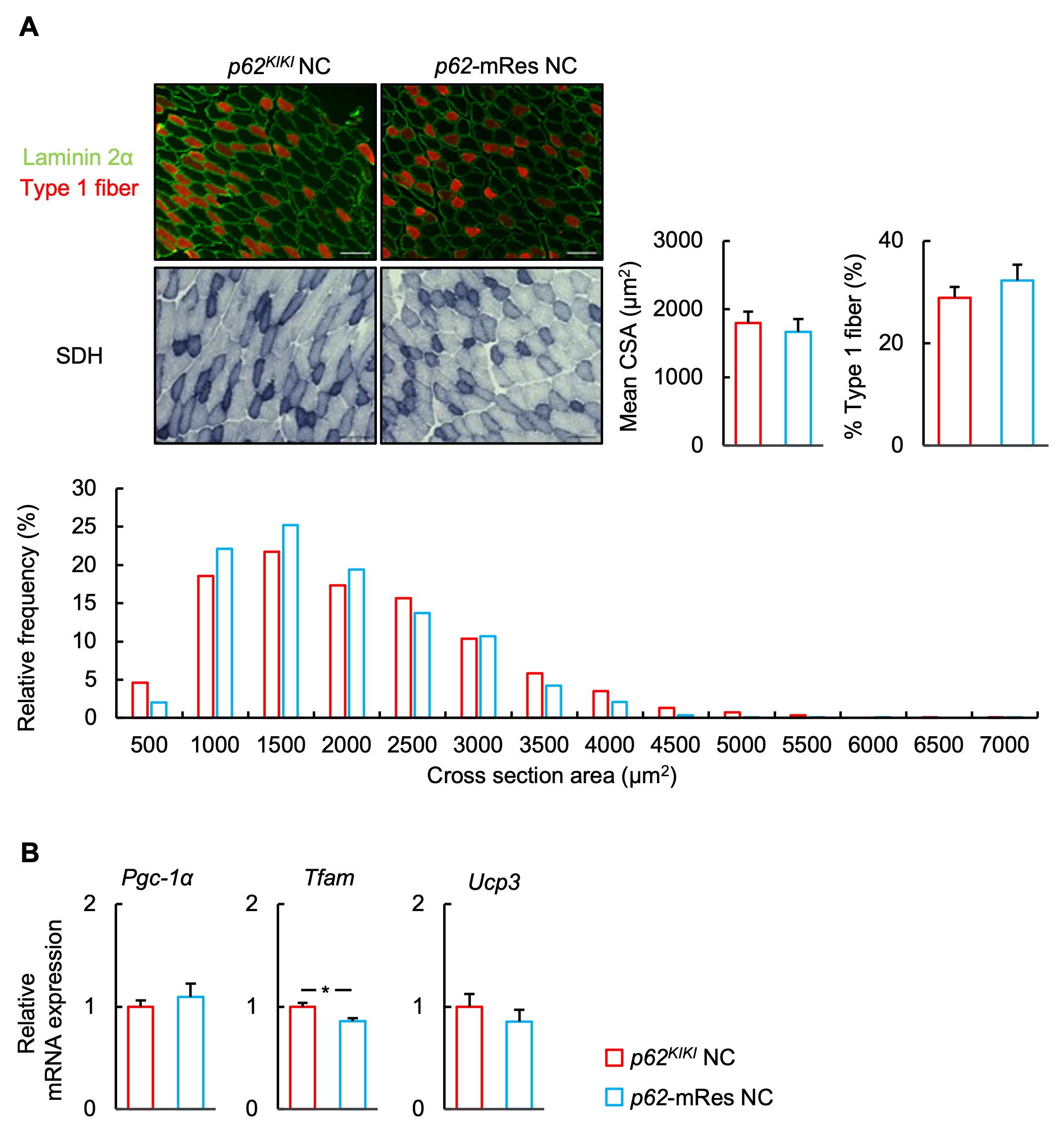

Supplement: Supplementary file 3 [file Image2.JPEG]

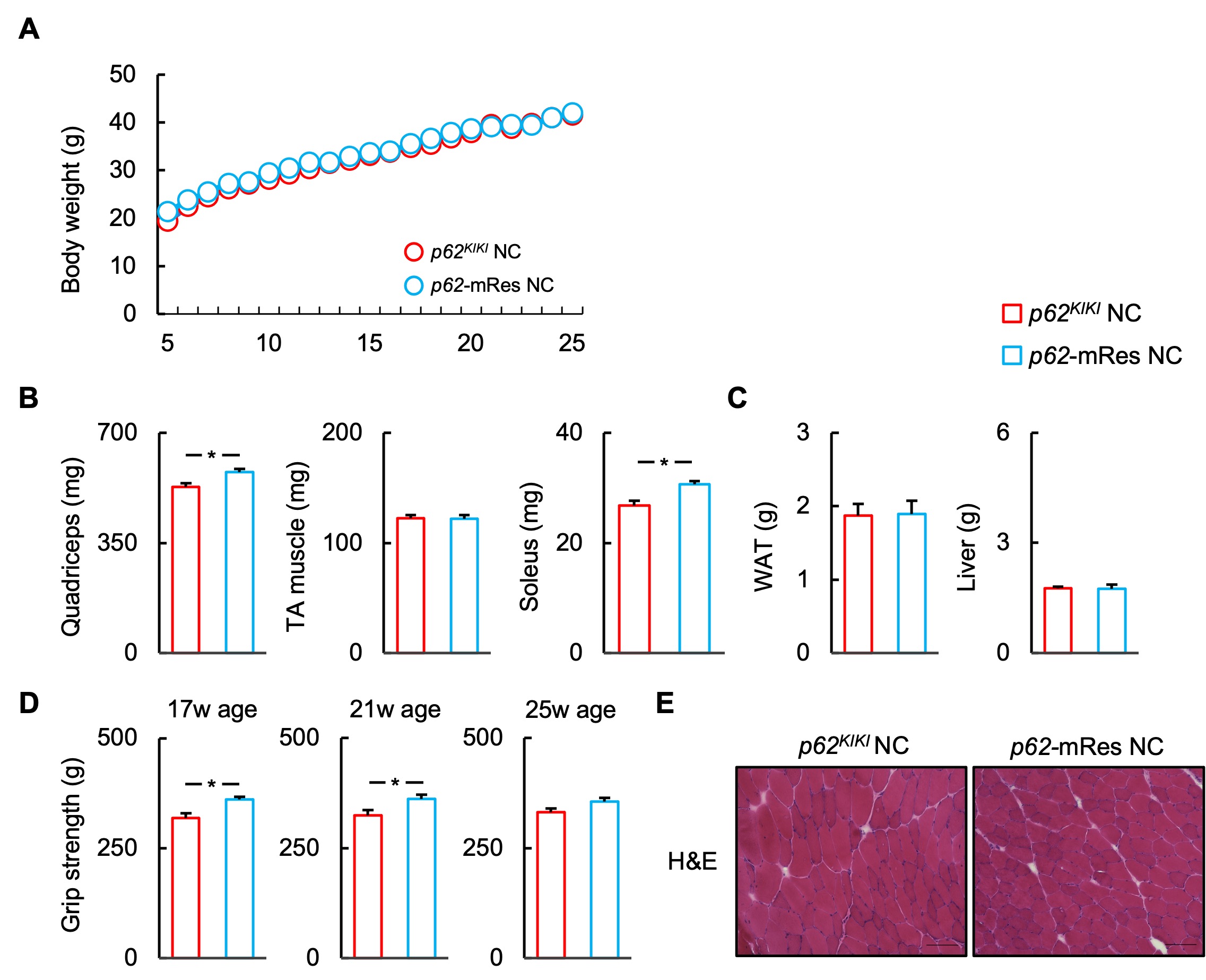

Supplement: Supplementary file 4 [file Image1.jpg]
